# Down-Regulation of Platelet Surface CD47 Expression in *Escherichia coli* O157:H7 Infection-Induced Thrombocytopenia

**DOI:** 10.1371/journal.pone.0007131

**Published:** 2009-09-22

**Authors:** Ya-Lan Guo, Dan-Qing Liu, Zhen Bian, Chen-Yu Zhang, Ke Zen

**Affiliations:** Institute for Virology, State Key Laboratory of Pharmaceutical Biotechnology, School of Life Sciences, Nanjing University, Jiangsu, People's Republic of China; Columbia University, United States of America

## Abstract

**Background:**

Platelet depletion is a key feature of hemolytic uremic syndrome (HUS) caused by Shiga toxin-producing *Escherichia coli* (STEC) infection. The mechanism underlying STEC-induced platelet depletion, however, is not completely understood.

**Methodology/Principal Findings:**

Here we demonstrated for the first time that platelet surface expression of CD47 was significantly decreased in C57BL6 mice treated with concentrated culture filtrates (CCF) from STEC O157:H7. STEC O157:H7 CCF treatment also led to a sharp drop of platelet counts. The reduction of cell surface CD47 was specific for platelets but not for neutrophil, monocytes and red blood cells. Down-regulation of platelet surface CD47 was also observed in isolated human platelets treated with O157:H7 CCF. Platelet surface CD47 reduction by O157:H7 CCF could be blocked by anti-TLR4 antibody but not anti-CD62 antibody. Down-regulation of platelet surface CD47 was positively correlated with platelet activation and phagocytosis by human monocyte-derived macrophages. Furthermore, the enhanced phagocytosis process of O157:H7 CCF-treated platelets was abolished by addition of soluble CD47 recombinants.

**Conclusions/Significance:**

Our results suggest that platelet CD47 down-regulation may be a novel mechanism underneath STEC-induced platelet depletion, and that the interactions between CD47 and its receptor, signal regulatory protein α (SIRPα), play an essential role in modulating platelet homeostasis.

## Introduction

Shiga toxin (Stx)-producing *Escherichia coli* (STEC) have been widely reported to be associated with cases of hemolytic uremic syndrome (HUS) [1], [2]. Although thrombocytopenia is a major feature of HUS, the mechanism by which the platelets are depleted in HUS is unclear. Previous studies indicated that platelet activation might be an important factor for thrombocytopenia since expression of platelet-derived products such as platelet factor 4 [3] and soluble P-selectin [4] were elevated during acute HUS. The plasma from patients with HUS also increased aggregation of normal platelets from healthy subjects. As possible causal factor of HUS, Stx1 and Stx2, are representatives of AB class of bacterial exotoxins [5]. For example, Stx can directly bind to human platelets via globotriaosylceramide (Pk antigen) and a novel platelet glycosphingolipid [6], and such binding may contribute to platelet activation and microthrombus formation observed in HUS. The toxin has also been identified in the kidney of HUS patients [7] and is cytotoxic for renal endothelial and epithelial cells [8], [9]. Moreover, animal models have reproduced aspects of HUS using wild-type bacteria that produced the toxin [10], [11], [12] or purified toxin [13], [14]. Culture filtrates from STEC were found to induce platelet-aggregating activity [15] although the experiments with purified Shiga toxin showed controversial results in platelet aggregation or P-selection expression [16], [17]. HUS-associated Shiga toxins were found to promote endothelial-cell secretion and impair ADAMTS13 cleavage of unusually large von Willebrand factor multimers [18]. Other STEC secreted components such as LPS also play a significant role in developing the aspects of HUS such as platelet activation and thrombocytopenia [19].

Serving as an integrin-associated protein and a self-recognition marker [20], [21], [22], [23], CD47 has been implicated in depletion of apoptotic cells and aging cells [21], [24]. Olsson et al [25] previously showed that platelet homeostasis was modulated by platelet CD47 under both normal condition and passive immune thrombocytopenia. The role of interactions between CD47 and its ligand, signal regulatory protein α (SIRPα), in regulating the clearance of platelets or other apoptotic cells by macrophages was also reported previously [26], [27], [28]. However, the alteration of platelet CD47 expression and its role in STEC infection-induced platelet depletion remains unclear.

In the present study, we demonstrate that platelet surface CD47 expression is specifically reduced in mice treated with concentrated STEC O157:H7-secreted products (CCF) and the effect of O157:H7 CCF is likely toll like receptor (TLR)-dependent. Down-regulation of platelet CD47 is positively correlated with an increase of platelet activation and aggregation, as well as the phagocytosis of platelets by macrophages.

## Materials and Methods

### Bacterial Strains and Reagents

EHEC O157:H7 (strain 99G144) was derived from an outbreak of hemolytic-uremic syndrome (HUS) in Xuzhou, Jiangsu, China in 1999. Toxin-negative *E. coli* O157:H19 (strain 99A041) was used as a control [29]. STEC isolates were serotyped using antisera against E-coli O antigens 1 to 173 and H antigens 15 to 56. PCR results against four major virulence genes *Stx1, Stx2, EaeA* and *Hly* have demonstrated that strain 99G144 is a *Stx2-eaeA-hly* type strain, while strain 99A041 is negative for signs of *Stx1, Stx2, eaeA*, and *hly* virulence genes. The Stx production was tested by using the Vero cell cytotoxicity assay and a commercial latex agglutination assay. Rat anti-mouse CD47 affinity purified mAb (clone miap301) was obtained from BD Biosciences (San Diego, CA). Anti-human TLR4 and TLR9 antibodies were obtained from Imgenex (San Diego, CA). Inhibitory mouse anti-human CD47 mAb (C5D5) was used as previously described [30]. Mouse Anti-Human CD61 mAb (Clone Y2/51) was obtained from DAKO (Carpinteria, CA).

### Preparation of concentrated culture filtrates (CCF) from STEC

STEC strains were grown overnight in Tryticase soy broth (Difco Laboratories, Detroit, MI) at 37°C with shaking (180 rpm), supernatants were filtered through 0.22 µm pore-diameter filters (Millipore) [31], and concentrated to 3-fold higher concentration before used.

### Animal procedure

6–8 weeks male C57BL6 mice (Jackson Laboratory, Bar Harbor, ME) were housed with free access to water and food in a specific pathogen-free facility. All animal care and handling procedures were carried out in accordance with the National Institute of Health's Guide for the Care and Use of Laboratory Animals and approved by the Institutional Review Board of Nanjing University, Nanjing, China. Mice were randomly divided into three groups. Group 1 (n = 6) included control mice (1 ml of saline was injected intraperitoneally); in group 2 (n = 8), 1 ml of CCF from O157:H7 (strain 99G144) was administered intraperitoneally on day 1 and day 2; in group 3 (n = 6), 1 ml of CCF from O157:H19 (strain 99A041) was administered on day 1 and day 2. All mice were sacrificed on day 4 after blood and urine sampling. Complete blood count and the measurement of lactic dehydrogenase (LDH) level were performed. CD47 knockout mice (CD47^−/−^) obtained from Jackson Laboratory served as a control for CD47 measurement.

### Cell isolation and labeling

Isolation and labeling of platelets were done as previously described [25] with a few modifications. In brief, anticoagulation buffer contained the following: 130 mM trisodium citrate, 10 mMEDTA, 10 mM theophylline, and 2 µg/mL carbacyclin. Buffered saline glucose citrate (BSGC) contained 110 mM NaCl, 14 mM trisodium citrate, 10 mM Na_2_HPO_4_-7H_2_O, 2 mM KH_2_PO_2_, 1 mM EDTA, 10 mM glucose, and 1 µg/mL carbacyclin, pH 6.8. For mouse platelets, blood was obtained by cardiac puncture using a syringe containing anticoagulant buffer. The blood was further diluted to a total volume of 1.5 mL in BSGC followed by centrifugation (300 *g*, 3 min). Platelet-rich plasma (PRP) fractions were collected, combined, and re-suspended in BSGC to a concentration of 5×10^8^ platelets/mL. Isolated cells were identified as platelets by labeling with anti-CD41 phycoerythrin (PE)-conjugated antibody (Immunotech). 2.5 µM CMFDA (Molecular Probes) was added to the platelets and incubated for 30 minutes at room temperature. Platelets were then centrifuged (1200 *g*, 10 min) to remove the free dye. Human platelets were isolated from periphery blood of healthy volunteers. The present study was approved by the Institutional Review Board of Nanjing University, Nanjing, China. Written informed consent was obtained from each participant. Human monocytes, neutrophils (PMNs) and red blood cells (RBCs) were isolated as previously described [32]. To prepare monocyte-derived macrophages (MDMs), isolated monocytes were cultured in DMEM/10% FCS supplemented with macrophage colony-stimulating factor (M-CSF) for 6 days [33]. Mature macrophages (3×10^5^/well) were then plated in 24-well tissue culture plates and allowed to adhere for 2 h. After removal of non-adherent cells, the cells were then cultured in DMEM/10% FCS without M-CSF for 24 h before use in phagocytosis assays. All the protocols and procedures were approved by Nanjing University Research Ethics Board.

### Immunofluorescence labeling and flow cytometric analysis

Blood was collected from a mouse tail vein and was diluted in PBS with 5 mM EDTA. Cells were then incubated with rat anti-CD47 monoclonal antibody (Clone miap301), followed by FITC-conjugated secondary antibody, and phycoerythrin-conjugated anti–mouse CD61 for 30 minutes on ice. After washing off unbound antibody, the samples were analyzed using FACscan flow cytometry and CellQuest software (BD Biosciences). Platelets were distinguished, based on the cell size and CD61 expression.

### Platelet adhesion

For platelet adhesion, commercial obtained fibrinogen (FBG) and purified SIRPα-GST chimera were immobilized onto 96-well plates prior to adhesion assays. SIRPα-GST chimera and GST protein (served as control) were prepared as previously described [32]. Fluorescently labeled platelets were then added into plates and incubated for 1 h at 37°C. After three washes, fluorescence intensity of each well was measured by a fluorescence plate reader (Molecular Devise) and cell adhesion was presented as percentage of total applied cells.

### Phagocytosis of platelets by monocyte-derived macrophages

Platelets were incubated with anti-platelet CD61 antibody for 20 minutes at room temperature for opsonization. The cells were then washed with HBSS and re-suspended in DMEM/10% FCS. Platelets (3×10^7^/per well) were then added, and the plates were centrifuged at 200 *g* for 1 min to establish contact between macrophages and platelets. Following incubation for 30 min at 37°C under 5% CO_2_, the macrophages were washed three times with HBSS. To remove non-ingested platelets, the macrophages were treated with 0.5 mM EDTA and 0.05% trypsin in PBS for 5 min. The ingested platelets by macrophages were analyzed by FV1000 confocal fluorescence microscope equipped with software system (Olympus). Percentage of phagocytosis was calculated as the fraction of macrophages with ingested platelets of the total number of macrophages analyzed in 6–8 random selected fields.

### Data analysis

Statistical analysis was performed by using Student *t* test for paired samples. All results are expressed as mean±SD. Values of *p*<0.05 were considered statistically significant.

## Results

**Figure 1:**
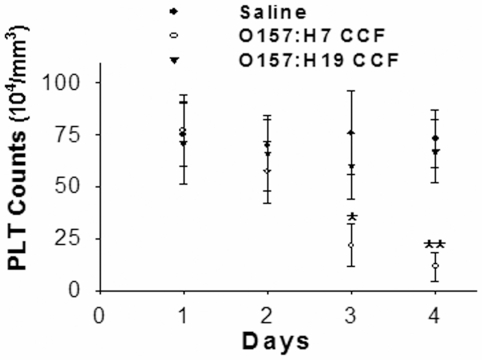
Platelet depletion in C57BL6 mice administered with concentrated culture filtrates (CCF) of STEC O157:H7 (strain 99G144). Note that platelet number was rapidly decreased in mice treated with O157:H7 (strain 99G144) CCF (n = 8) compared to that in mice treated with saline (n = 6) or CCF from O157:H19 (strain 99A041) (n = 6). *, p<0.05, **, p<0.01.

In our previous study, an EHEC O157:H7 (strain 99G144) was derived from an outbreak of HUS in Xuzhou, Jiangsu Province, China in 1999. PCR results against four major virulence genes *Stx1, Stx2, eaeA* and *hly* have shown that strain 99G144 is a *Stx2-eaeA-hly* type strain [29]. To assess the ability of STEC O157:H7 (strain 99G144) to induce HUS-like syndrome in animal, we prepared the concentrated culture filtrates (CCF) from culture medium of EHEC strain 99G144 and Shiga toxin-negative O157:H19 (strain 99A041) (served as a control), respectively, and then intraperitoneally injected 0.5 mL/per day of these CCFs into C57BL6 mice. As shown in Figure 1, mice treated with CCF of strain 99G144 had a sharp drop of platelet count on day 4 post-injection compared to mice that injected with saline or strain 99A041 CCF. In addition, significant injure particularly bleeding in multiple organs including kidney, eye and intestine was observed in mice treated with the CCF of strain 99G144 (data not shown). The results implicated that C57BL6 mice treated with STEC O157:H7 CCF suffered a HUS-like symptom.

**Figure 2:**
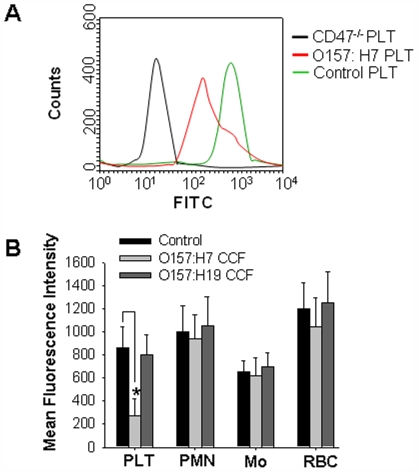
Reduction of platelet surface CD47 expression in mice treated with CCF from STEC O157:H7 (strain 99G144). A, CD47 expression on platelet surfaces measured by flow cytometry. Cells derived from CD47^−/−^ mice served as a negative control in CD47 immunofluorescence labeling and measurement by flow cytometry. B, CD47 expression levels on the surfaces of platelets (PLT), neutrophils (PMN), monocytes (MO), and red blood cells (RBC), respectively. Shiga toxin-negative strain 99A041 (O157:H19) was used as a control for STEC O157:H7 (strain 99G144). Data were presented as mean±SD of three independent experiments. *, p<0.05.

We next assessed the expression level of CD47 on the surfaces of platelets, neutrophils (PMNs), monocytes, and red blood cells (RBCs), respectively. Surprisingly, we found that, on day 3 post-injection, strain 99G144 CCF treatment strongly decreased CD47 expression on platelet cell surface measured by FACscan flow cytometry (Figure 2A). In contrast, CD47 expression levels on the surfaces of other cell types in mouse circulating blood stream, such as PMNs, monocytes, and RBCs, were not affected by O157:H7 CCF treatment (Figure 2B). Served as a control, treatment with CCF from Shiga toxin-negative O157:H19 (strain 99A041) did not affect platelet surface CD47 expression level.

**Figure 3:**
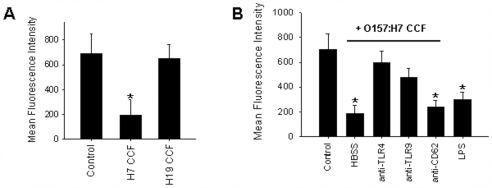
Reduction of CD47 surface expression level on isolated human platelets by STEC O157:H7 (strain 99G144) CCF. A, strain 99G144 CCF but not O157:H19 CCF decreases CD47 surface expression. B, Reduction of CD47 expression levels by strain 99G144 CCF in the presence of various antibodies at concentration of 25 µg/ml each. Data were presented as mean±SD of three independent experiments. *, p<0.05.

The effect of STEC O157:H7 CCF on platelet surface CD47 expression was further determined using isolated human platelets. For these experiments, platelets were isolated from periphery blood of healthy donors, washed with HBSS containing EDTA, and then incubated with CCF from O157:H7 (strain 99G144) or O157:H19 (strain 99A041) at 37°C under 5% CO_2_. As shown in Figure 3A, platelet surface CD47 expression was reduced by treatment with strain 99G144 CCF but not strain 99A041 CCF, implicating that the component(s) in strain 99G144 CCF can directly interact with platelets and down-regulate platelet CD47 expression. Based on the fact that STEC strain 99G144 is a Stx2-type strain [29] and we did detect significant amount of stx2 in CCF of strain 99G144 but not strain 99A041 (data not shown), the reduction of platelet surface CD47 by STEC strain 99G144 CCF might be partially due to direct interaction between Stx2 and platelets. Since it has been reported that toll like receptors (TLRs) [19] and CD62 [34] were involved in themocypernia induced by STEC-derived products, we next tested whether TLRs and CD62 play a role in platelet surface CD47 reduction and platelet depletion induced by STEC strain 99G144 CCF. As shown in Figure 3B, functional anti-TLR4 antibody strongly blocked the effects of strain 99G144 CCF on platelet surface CD47 expression while anti-CD62 antibody showed no effect. Antibody against TLR9 also reduced the effect of STEC strain 99G144 CCF on platelet surface CD47 expression, albeit at much lower intensity. The results implicated that TLR4-mediated signal pathway might be involved in the modulation of platelet surface CD47 expression by strain 99G144 CCF treatment. This conclusion was also supported by directly treating platelets with lipopolysaccharide (LPS), a TLR4 activating substance [35]. The platelet surface CD47 expression was significantly decreased by LPS treatment (Figure 3B).

**Figure 4:**
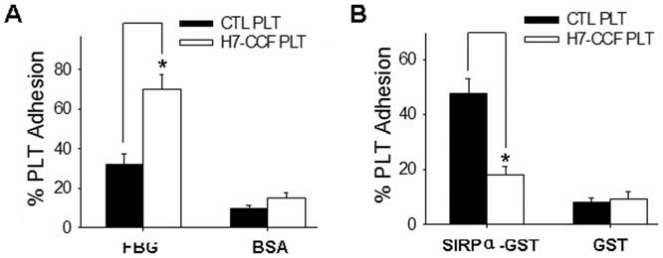
Down-regulation of platelet surface CD47 expression level positively correlates with activation of platelets. The adhesion of platelets to immobilized fibrinogen (FBG) (A) and SIRPα extracellular domain recombinant (SIRPα-GST) (B) was measured by an *in vitro* binding assay. BSA and GST were used as the controls for FBG and SIRPα-GST in binding assays, respectively. Data were presented as mean±SD of three independent experiments. *, p<0.05.

To examine the functional status of platelets after treatment with the CCF of STEC O157:H7, isolated human platelets incubated with or without strain 99G144 CCF were further allowed to adhere to 96-well tissue culture plates coated with fibrinogen (FBG) or soluble recombinant of SIRPα extracellular domain (SIRPα-GST). It has been known when platelets are activated, there are more platelets adhere to immobilized FBG [36], [37]. As shown in Figure 4A, adhesion of platelets treated with strain 99G144 CCF to immobilized FBG was significantly higher than that of non-treated platelets, suggesting that platelets treated with strain 99G144 CCF were activated. These same platelets treated with strain 99G144 CCF, however, showed a decreased adhesion to immobilized SIRPα-GST recombinant compared to non-treated platelets, confirming that platelets treated with strain 99G144 CCF have less functional SIRPα-binding CD47 on their surfaces (Figure 4B). Together, these results demonstrate that platelets treated with strain 99G144 CCF are in activated form and contain less cell surface CD47.

**Figure 5:**
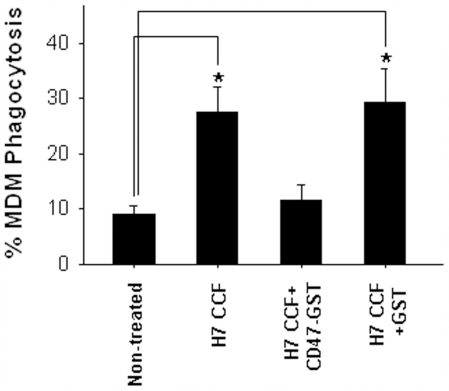
Enhanced phagocytosis of O157:H7 CCF-treated platelets by human monocytic-derived macrophages. Percentage of phagocytosis was calculated as the fraction of macrophages with ingested platelets of the total number of macrophages analyzed in 6–8 random selected fields per slide. Soluble CD47 extracellular domain recombinant and GST only were added at 40 µg/ml each. Data were presented as mean±SD of three independent experiments. *, p<0.05.

We next determined the phagocytosis of platelets treated with or without strain 99G144 CCF by human monocyte-derived macrophages (MDMs). Platelets were treated with anti-platelet IgG for opsonization. In separate experiments, incubation was performed in the presence of inhibitory anti-SIRPα antibody or soluble CD47 extracellular domain recombinant (CD47-GST). As shown in Figure 5, after 3 h incubation, phagocytosis of strain 99G144 CCF-treated platelets by MDMs was significantly higher than that of non-treated platelets or platelets treated with strain 99A041 CCF. In fact, internalization of non-treated platelets by MDMs was very low. The enhanced uptake of strain 99G144 CCF-treated platelets by MDMs, however, was abolished by addition of soluble CD47 extracellular domain recombinant. These results argue that down-regulation of CD47 expression level on platelet surfaces is positively correlated with platelet depletion by phagocytes such as macrophages, and that the interactions between platelet CD47 and macrophage SIRPα play a critical role in regulating platelet depletion in STEC infection-induced thrombocytopenia.

## Discussion

In the present study, we reported for the first time that platelet surface CD47 reduction is a critical step of platelet depletion in STEC-induced HUS. This conclusion was supported by the data derived from both *in vivo* and *in vitro* experiments. First, co-occurrence of platelet depletion and down-regulation of platelet surface CD47 was observed in STEC O157:H7 infected mice. Reduction of CD47 expression level was likely specific for platelets since other cells in mouse blood stream had no alteration of surface CD47 after treatment. Second, we confirmed the effect of STEC O157:H7 CCF on reduction of platelet surface CD47 and showed the direct role of STEC strain 99G144 CCF using isolated human platelets. Furthermore, we found that the reduction of platelet surface CD47 expression by strain 99G144 CCF could be blocked by anti-TLRs antibodies, implicating that TLRs-mediated signal downstream might play a significant role in regulating the interactions of platelets with the virulent components of STEC strain 99G144. Third, employing in vitro binding assay, we showed that the platelets treated with secreted products from STEC O157:H7 (STEC strain 99G144) were in active condition (Figure 4A) and contained less functional ligand-binding CD47 on the surface (Figure 4B). Finally, we demonstrated that activated platelets after STEC O157:H7 CCF treatment could be rapidly removed by macrophages through phagocytosis, and that the phagocytosis of platelets by macrophages could be blocked by soluble CD47 extracellular domain recombinant (Fig. 5). These results suggest that the binding interaction between CD47 and its cellular liagnd, signal regulatory protein α (SIRPα), serves as a critical negative regulator for platelet depletion by phagocytes such as macrophages.

Previous studies have shown that Shiga toxins producing *Escherichia coli* are the main cause of the hemolytic uremic syndrome [10], [11], [12], [38]. However, although our data implicated that Shiga toxins might directly interact with platelets and play a role in platelet surface CD47 reduction, the exact components in STEC strain 99G144 CCF that affect platelet CD47 expression remain unclear. Besides Stx2, LPS might also be the candidates in down-regulating platelet cell surface CD47 expression. It also remains unknown why infection by STEC O157:H7 (strain 99G144) selectively reduces platelet surface CD47 expression but not other circulating cells such as monocytes, neutrophils and red blood cells. The possible reason underneath this specific platelet CD47 reduction may be that platelets express unknown receptor(s) for virulent factors such as Stx2 or LPS.

In summary, our results indicate that CD47 is a critical molecule that negatively regulates STEC-induced activation and depletion of platelets. Down-regulation of CD47 level on platelet surfaces by virulent factors of STEC O157:H7 is likely through TLRs-mediated signaling pathway. CD47 down-regulation on platelet surfaces results in a loss of the negative regulatory mechanism initiated by CD47-SIRPα interactions [21], which in turn, leads to platelet activation and consequent phagocytosis of platelets by macrophages or other SIRPα-positive immune cells. Since soluble CD47 extracellular domain recombinant can block the enhanced phagocytosis of STEC virulent factors-treated platelets by macrophages, CD47 may be a novel therapeutic targets aiming at attenuating and preventing STEC infection-induced platelet depletion.
